# Lipid changes within the epidermis of living skin equivalents observed across a time-course by MALDI-MS imaging and profiling

**DOI:** 10.1186/s12944-015-0089-z

**Published:** 2015-08-05

**Authors:** Christopher A. Mitchell, Heather Long, Michael Donaldson, Simona Francese, Malcolm R Clench

**Affiliations:** Biomedical Research Centre, Sheffield Hallam University, Howard Street, Sheffield, S1 1WB UK; Stiefel A GSK Company, GlaxoSmithKline, Stockley Park West, Uxbridge Middlesex, UB1 1BT UK

**Keywords:** MALDI-MSI, MALDI, Mass spectrometry imaging, MSI, Lipids, Living skin equivalents

## Abstract

**Background:**

Mass spectrometry imaging (MSI) is a powerful tool for the study of intact tissue sections. Here, its application to the study of the distribution of lipids in sections of reconstructed living skin equivalents during their development and maturation is described.

**Methods:**

Living skin equivalent (LSE) samples were obtained at 14 days development, re-suspended in maintenance medium and incubated for 24 h after delivery. The medium was then changed, the LSE re-incubated and samples taken at 4, 6 and 24 h time points. Mass spectra and mass spectral images were recorded from 12 μm sections of the LSE taken at each time point for comparison using matrix assisted laser desorption ionisation mass spectrometry.

**Results:**

A large number of lipid species were identified in the LSE via accurate mass-measurement MS and MSMS experiments carried out directly on the tissue sections. MS images acquired at a spatial resolution of 50 μm × 50 μm showed the distribution of identified lipids within the developing LSE and changes in their distribution with time. In particular development of an epidermal layer was observable as a compaction of the distribution of phosphatidylcholine species.

**Conclusions:**

MSI can be used to study changes in lipid composition in LSE. Determination of the changes in lipid distribution during the maturation of the LSE will assist in the identification of treatment responses in future investigations.

**Electronic supplementary material:**

The online version of this article (doi:10.1186/s12944-015-0089-z) contains supplementary material, which is available to authorized users.

## Background

Living skin equivalent (LSE) models are an important resource for skin research and are commonly employed as an alternative to the use of *ex-vivo* mammalian tissue or *in-vivo* experiments in the pharmaceutical and cosmetics industries. A wide variety of the skin models are commercially available and can be manufactured in-house with features tailored to a particular need [1].

As a major component of skin, lipids play important roles in cellular functions and are agents within cell signalling cascades. Ceramide metabolism for example is known to be highly compartmentalized, since, because ceramides are highly hydrophobic; they tend to reside in the specific membranes where they are generated, unless transported elsewhere [2, 3]. Psoriasis and atopic dermatitis skin lesions on inspection usually present a deficiency in ceramide and water content [4]. The progression of proliferation and differentiated states in skin can be correlated with the content of ceramides and glycophospholipids in a targeted manner, supporting both biomarker discovery within skin pathology and pharmacodynamic investigations.

Mass spectrometry imaging (MSI) is a relatively new technique for the analysis of compounds in the skin, it offers great promise since it is able to detect and plot the distribution of multiple compounds simultaneously in a label-free manner [5]. In addition, by employing tandem MS analysis, the detailed structure of metabolite molecules, such as lipids, can be identified directly on the tissue sections; thus, it can be confirmed that the observed mass signals are derived from the molecules of interest. MSI has been previously used to analyse human skin biopsies taken from patients with eczema. In this case a role for glycosphingolipids in disease pathogenesis was observed [6].

We have previously reported the use of MALDI-MSI to study the disposition of a topical compound absorbed in porcine skin [7]. Since then however there have been few publications, dealing with skin and MSI analysis. The correlation of skin blanching and percutaneous absorption of a glucocorticoid receptor agonist was reported by Marshall *et al.*, [8]. Prideaux *et al.*, [9] reported the examination of xenobiotic compounds in skin tissue sections by indirect MALDI-MSI and more recently Hart *et al.*, [10] analysed *ex-vivo* human skin via MSI. Enthaler *et al.*, [11] demonstrated that small endogenous species such as cholesterol sulphate can be mapped by MSI in the stratum corneum. Recently MSI was used for the examination of imipramine absorption in a LSE [12]. Although the metabolism of the drug was examined *i.e.* drug pharmacokinetics, changes to the skin in response to the pharmaceutical compound *i.e.* pharmacodynamic responses were not investigated.

Here we report a study of the distribution of lipids within LSE and an examination of cell differentiation in the LSE across a time-course. Multivariate statistical analysis has been employed to discriminate inter-spectral ion changes of significance so that the distribution of projected ions could be imaged. The generated datasets will provide a reference point to examine *in-vitro* disease equivalent models and lipid responses to treatment in future investigations.

## Results

### MALDI-MS and MALDI-MS/MS analysis of LSE sections

**Fig. 1:**
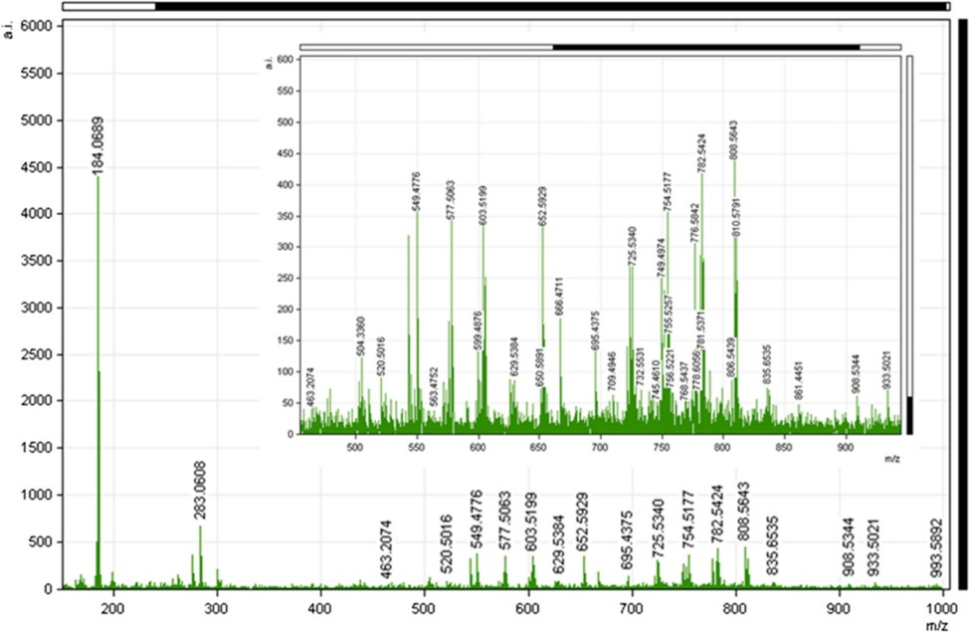
Positive ion MALDI Mass spectrum of a LSE (24 h incubation) across the full thickness of the tissue, using α-CHCA/ANI as a matrix

A representative mass spectrum obtained from the untreated LSE 24 h incubation sample group is shown in Fig. 1. The spectrum shows the large number of lipid species present within the sample. Additional file 1: Table S1 shows the results of the database search of the accurate masses (5 ppm) of detected peaks. The lipid groups identified and reported in the table include free-fatty acids, glycerophosphonoethanolamines, glycerophosphocholine, sphingolipids and triacylglycerols. Phosphatydlcholine (PC), sphingomyelin (SM) and lyso phosphatydlcholine (LPC) species appear to be most dominant in the average spectra. PC lipids were particularly populated between the *m/z* 670 and 900 range with intensity counts approximately 4 times greater than the other low abundant signals at the lower mass ranges. Many of the detected compounds including PC 34:2 (*m/z* 780.5 M + Na^+^), PC 36:2 (*m/z* 786.6 M + H), PC 36:1 or PE 39:1 (*m/z* 810.5 M + Na^+^) were previously identified in *ex-vivo* human skin also [10]. The dominance of phospholipids in the spectra is probably due to their biological abundance in tissue; the fixed positive charge from the choline head-group allows PC species to have strong ionisation efficiencies.

A common feature in the data, ion *m/z* 703.5 was tentatively assigned as sphingomyelin (SM) 34:1 from prior experimentation [10]. To further characterise this feature we observed the ion mobility drift time plot for the MALDI-IMS-MS profiles of control LSE tissue and a SM (d18:1/16:0) standard both in the presence of lithium salts and then without. A common drift-time value of 101 can be observed in Additional file 1: Figure S1a for the *m/z* 703.57 [M + H]^+^ ion and the *m/z* 709.56 [M + Li]^+^ ions respectively. Additional file 1: Figure S1b shows IMS-MS/MS data obtained from the peak at *m/z* 709.57*.* The MSMS spectrum for the selected precursor ion (*m/z* 709.57) gave product ions that were expected for the SM (d18:1/16:0) compound and also some that were assigned as arising from structural isomers *e.g. m/z* 236.2; which is known to represent the d16:1 long chain base of a ceramide structure rather than the d18:1 chain. Additional file 1: Table S1 indicates which of the tentatively identified lipids were verified via MSMS alone by comparison of MSMS spectra to authentic lipid standards and where ion mobility drift times were employed.

By manual examination of the full mass spectral data set it was possible to find instances where ceramides were not dominated by phosphocholine in the spectra. This appeared to correspond to the uppermost edge of the skin. There were common features in the spectra which appeared to be structural motifs of the ceramide lipid family, particularly *m/z* 264.2668 and 256.2599 (d18:1 side chains). A number of possible dehydrated ceramide features detected with the loss of H_2_O [M + H-H_2_O] via high accurate mass measurement; *i.e. m/z* 534.5 cer (d15:1/20:0) and *m/z* 520.2 ceramide 16 (N-paltimitoylsphingosine)*,* could be putatively identified. Some sphingolipid signals which are incorporated into higher structures were interrogated further in the imaging experiment.

Fatty acyls particularly monounsaturated species in the smaller mass range had a much lower signal to noise ratio. This low abundant phenomenon appears to be a common finding in skin models and has been previously reported by Thakoersing *et al.*, [13, 14]. Tentative identifications include oleic acid ([M + H]^+^, *m/z* 283.2620), linoleamide ([M + H]^+^, *m/z* 280.2655), acetic acid ([M + K]^+^, *m/z* 98.9828) and eicosenic acid ([M + H]^+^, *m/z* 311.2875) respectively. Isobaric hexadecadienal variant species at *m/z* 237.2175 were difficult to distinguish from each other through ion-mobility alone. Matrix clusters were abundant in the fatty acyl mass range.

As anticipated phosphatidylinositols (PI) and phosphatidylserines (PS) and cholesterol species were of low abundance in the spectrum as they are better ionised in the negative mode.

### Multivariate statistical analysis of spectra across time-course (4, 6 and 24 h)

**Fig. 2:**
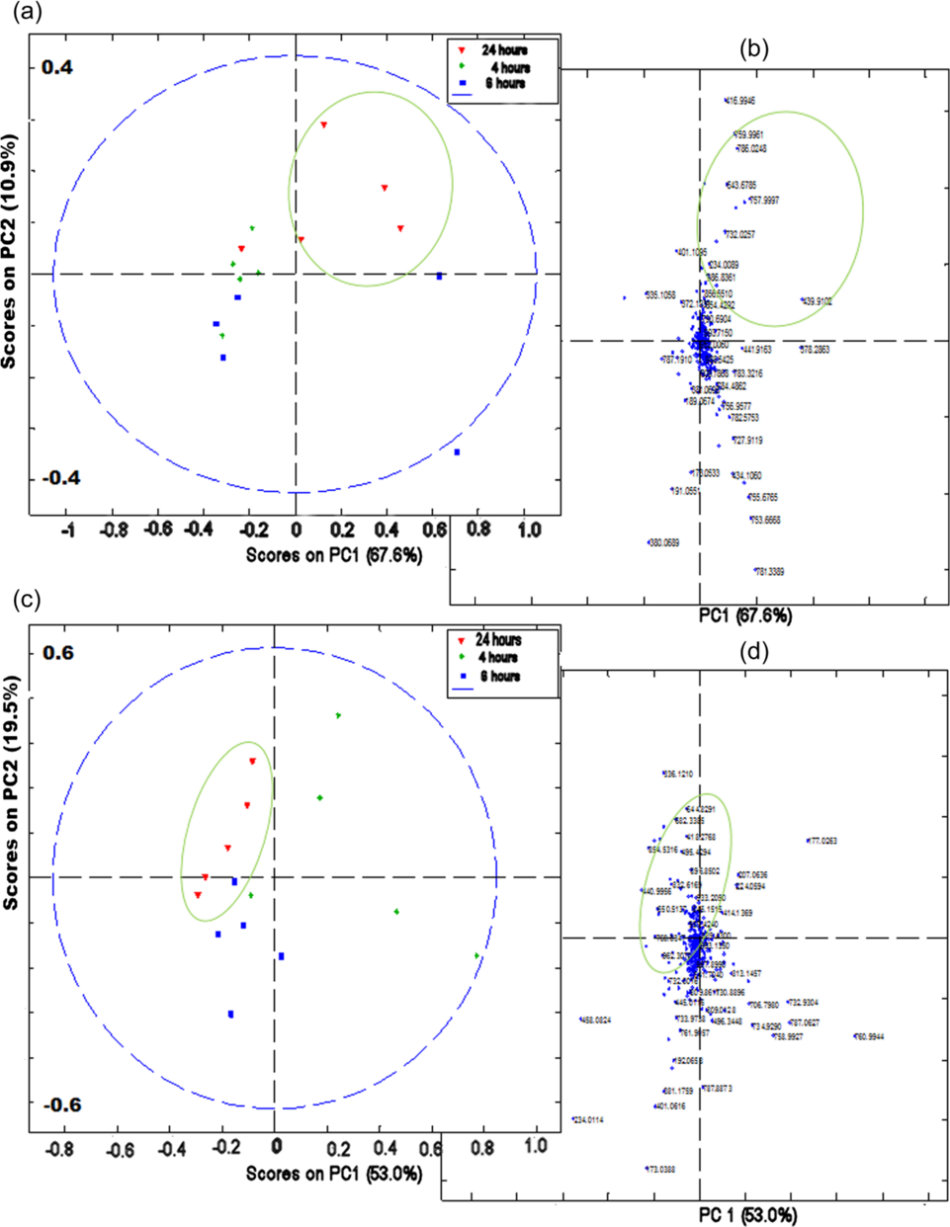
Principal component analysis of MALDI-MS spectra acquired from LSE. Score plots were generated showing groupings and variability between the 4, 6 and 24 h group spectra from the **a**) epidermis or **c**) dermis regions of the skin. The loading plots show a distribution of m/z spectra ion species which are contributors of grouping and variability in the **b**) epidermis or **d**) dermis between the time-course groups; the quadrant space in corresponding between the plots

Principle component analysis (PCA) scores and loadings plots were performed to identify changes that occurred in the LSE samples during the time course study. Figure 2 shows the PCA plots obtained from the analysis of the spectra taken from the epidermis alone: Fig. 2a, c shows the scores plot giving the extent of groupings and variability between the skin spectra at different time points. Figure 2b and d shows the loadings plot comprising of the spatial distribution of *m/z* spectral species in correspondence to the scores plot positions. Figure 2a and b show the corresponding scores and loadings plots for observable changes in epidermal regions; Fig. 2c, d for the dermis. The epidermis PCA scores plot (Fig. 2a) show an overall trend which is that; the 24 h group is more separated in the PCA space than the 4 and 6 h spectra groups. For the scores plots for in the dermal region however (Fig. 2c), the spectra seemed to associate more with each other as the time-course progressed; the 4 h group being the most distinct. In the loadings plot obtained from the epidermis data (Fig. 2b) it can be seen that *m/z* 759, 757 and 732 are major contributors to the observed variance. Examination of the dermis PCA loadings plot (Fig. 2d), suggests that *m/z* 760, 758, 732 and 734 appeared to distinguish the 4 h group within the lower right quadrants of the loadings plot.

### MALDI-MS Imaging analysis of LSE sections

MALDI-MSI-IMS images were acquired from LSE tissue derived from 3 biological samples incubated for 4, 6 and 24 h per experiment. The spatial resolution was set to 50 μm × 50 μm. Images were processed using Waters HDI software (Waters Corporation); which incorporates the added measurements of ion mobility.

**Fig. 3:**
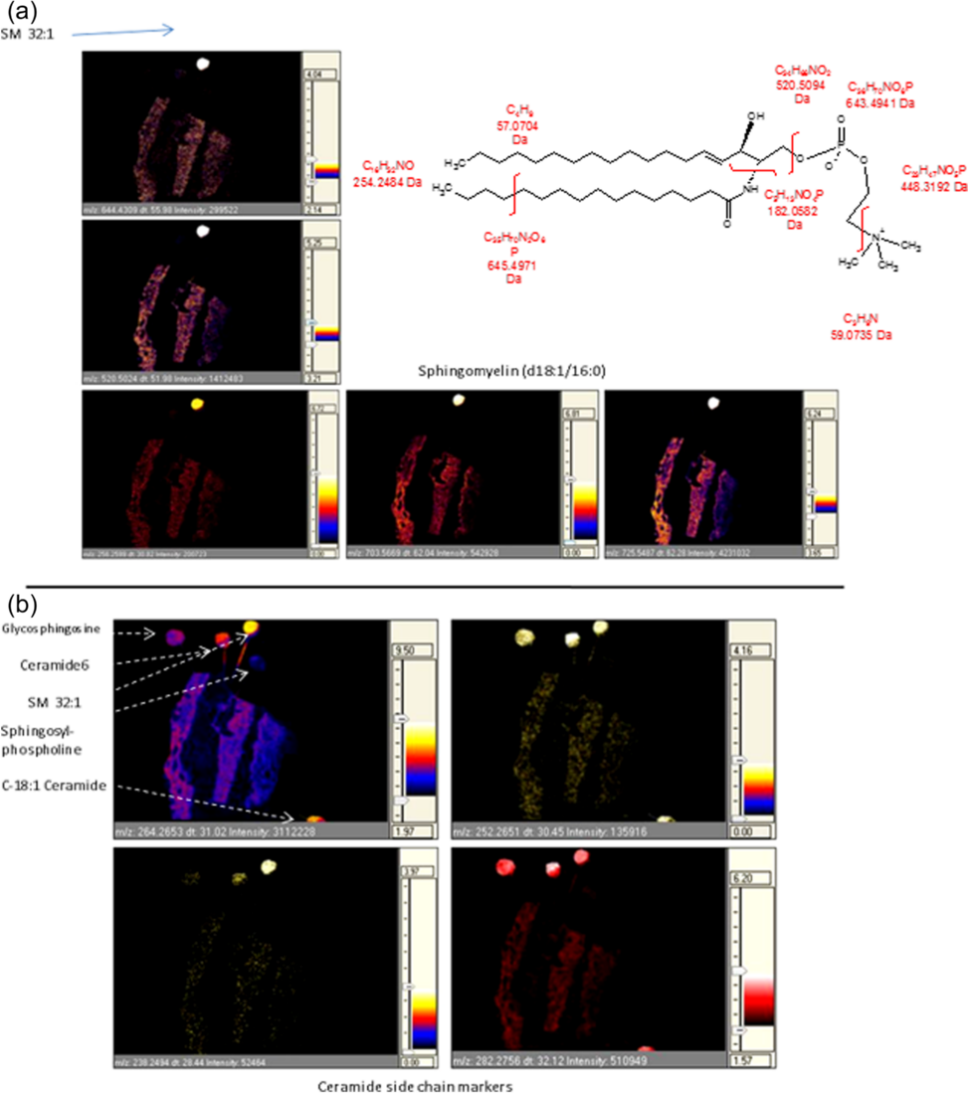
**a** MALDI-MSI-IMS image mapping the in-source fragment *m/z* ion species for SM (d18:1/16:0) in tissue and within a SM (d18:1/16:0) lipid standard. Particularly *m/z* 520.5024 is likely to be the N-paltimitoylsphingosine unit of the structure following neutral loss of cyclophosphane [H (HO)P (O) (OCH2CH2O)] *m/z* 183. The ion m/z 644.4309 correspond to a loss of trimethylamineN (Ch3) *m/z* 59 from the M + H adduct. Ion map for *m/z* 256.2599 represents the d18 side chain of the SM lipid structure. The approximate fragment conformations are depicted in the structural diagram. **b**. MALDI-MSI-IMS of commonly occurring fragment ions found within tissue and lipid standards representative of fatty acyl side chains

In the imaging experiments it was possible to gain further information using spotted lipid standards in the image path but just off the tissue. Figure 3a shows that some of the ions detected are fragment ions of larger species *e.g.* SM (d18:1/16:0). Here, *m/z* 520.5024 detected in tissue at a drift-time value of 51.98 is also observed as a fragment ion of the SM (d18:1/16:0) lipid standard. It can be assigned to loss of cyclophosphane [H (HO) P (O) (OCH_2_CH_2_O)] *m/z* 183, from the protonated SM species, forming N-paltimitoylsphingosphine. Similarly the *m/z* 644.4309 ion corresponds to a loss of trimethylamine N (CH_3_) *m/z* 59. The fragment ion *m/z* 256.2599 corresponds to the d18 side chain within the sphingolipid structure. When the images of the fragment species (*e.g. m/z* 520.5024 and 644.4309) were compared with the protonated and sodium adduct ion images (*m/z* 703.5669 and 725.5487) a similar distribution of signal was observed between images; the main difference being intensities as expected.

In addition, other structural ion markers could be visualised in multiple combinations of lipid standards and in tissue (Fig. 3b). The lipid standards in addition to SM d18:1/16:0 includedglycosphingosine, sphingosylphosphorylcholine (SPC), ceramide 6 (hexanoyl-D-erythro-D-sphingosine) and C18:1 ceramide (N-oleoyl-D-sphingosine). When mapping ion *m/z* 264.2653 a distribution of signal was visualised in tissue and abundantly within the ceramide 6, SM (d18:1/16:0) and C18:1 ceramide lipid spot standards, with moderate signal found in the glycosylsphingosine spot standard and trace signals in the SPC lipid spot. When mapping ions within the ceramide mass range, signals were visualised in the tissue and in particular spot standards in some instances, for the various sphingoid base lipid structures as shown in Additional file 1: Figure S2. Thus the spot standards could be used to support putative identifications using the side chain markers. Identification of some choline rich species was also supported in a similar way, particularly with the use of the PC (16:0/18:1(9Z)) and SM (d18:1/16:0) lipid spot standards.

**Fig. 4:**
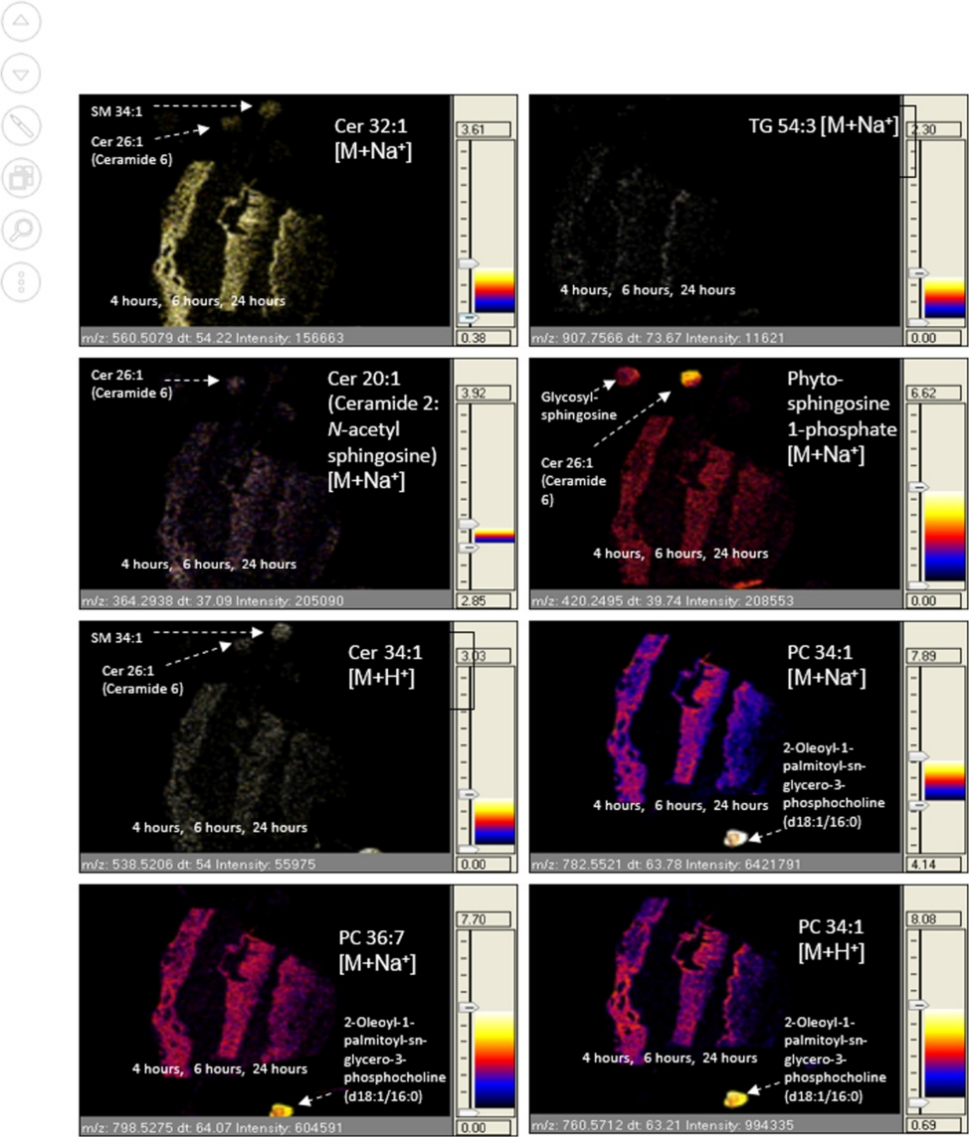
MALDI-IMS-MSI images mapping the protonated and sodiated ions for ceramides and PC species across skin equivalent tissue sections representing the three time-stages within the time-course study (4, 6 or 24 h incubation period [*from left to right*]) and across lipid standards. Images are at a spatial resolution of 50 μm × 50 μm and are normalised against the total ion count

Figure 4 shows MALDI-MS images of identified lipid compounds in the tissue. Trigylcerol (TG) 54:3 (*m/z* 907.7566 [M + Na]^+^) is a marker of the stratum corneum; its distribution can be observed at the outer most regions of the skin sections, as reported also by Hart *et al.*, [10]. The ceramide species when visualised appeared less intense generally in comparison to the PC lipid ion maps in the tissue. Some broad differences could be observed between the tissue sections (4, 6, 24 h) for most of the images. The distribution of the ceramide and PC ions in the 24 h sample appeared to be located primarily towards the top outer region of the tissue section, whereas in the 4 and 6 h skin sections, the signal is observed throughout the sections.

**Fig. 5:**
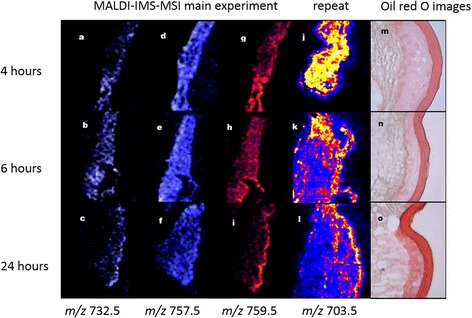
MALDI-IMS-MS images mapping the ions *m/z* 759.56, 757.54, 732.54 and 703.56 in skin equivalent tissue, at the different stages of a time course (4, 6 and 24 h respectively). **a** - (**c**) *m/z 732.54* at 4,6, and 24 h, (**d**)-(**f**) *m/z* 757.54 (**g**)-(**i**) *m/z* 759.56 at 4,6 and 24 h and (**j**)-(**l**) *m/z 703.56 at 4,6, and 24 h* . These signals were identified from the PCA data as being significant contributors of change in the spectra between the epidermis and dermis. Images are at a spatial resolution of 50 μm × 50 μm and are normalised against the total ion count. Optical images of Oil Red O stained sections of the tissue at the different stages of a time course (4, 6 and 24 h respectively) are also shown (**m**)-(**o**)

**Fig. 6:**
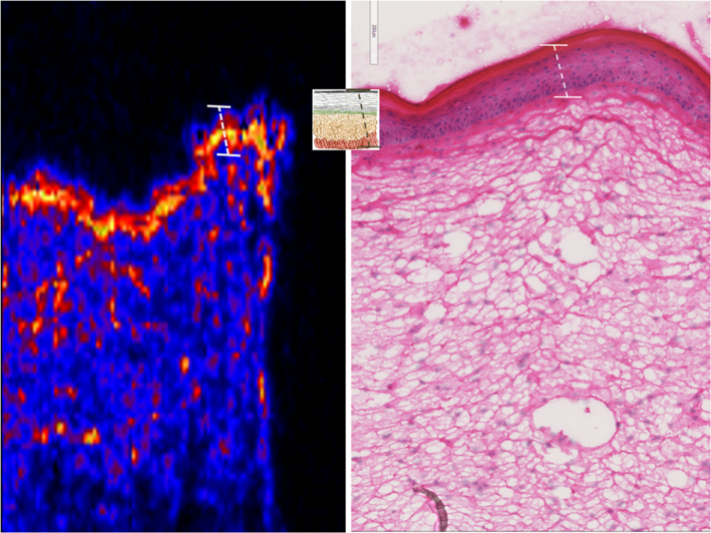
Haematoxylin & eosin stained skin section against a MALDI-MS map of skin sections focusing on the 24 h time-point, showing the potential of the 50 μm × 50 μm resolution to discern epidermal ultrastructural region

The PCA plots show ions which are major contributors to variances in the epidermis (Fig. 2a, c). Figure 5 shows MALDI-MS images for the distribution of the ion *m/z* 759.5592 which was projected to vary significantly between the dermis and epidermis from the PCA loadings plot. From these images some changes can be visualised: the distribution of the ion*m/z* 759.5592 changes with time; in the 24 h sample it is located primarily at the edge of the epidermis whereas in the 4 and 6 h tissue sections the signal is observed throughout the sections. This is also the case for the ions *m/z* 757.5411 and *m/z* 732.5531. These species have been tentatively identified via accurate mass measurement (Additional file 1: Table S1) to an error of 5 ppm. Repeat studies carried out on a different instrument gave the same findings; a distinct epidermal band can be seen when mapping select ions, particularly for the 24 h sample section. This is particularly shown for the ions *m/z* 759.5 and 703.5 ions. When directly comparing the Oil Red O stained sections across the time-course, noticeably some basal and granular epidermal layer changes can be seen in the later time-point. The epidermal region appears more compressed and dense in the 24 h sample, whereas the 4 h sample is thicker in appearance and less compact. Some lipid droplets can be found in the 24 h sample epidermis. Intriguingly the 24 h stratum corneum appears darker indicative of being a richer neutral lipid layer.In all cases a stratified epidermis could be visualised as a red band across the outer region of the skin. This stratified feature can also be shown as a dark purple edge in the haematoxylin and eosin stained sections across the time-course (Fig. 6).

In order to confirm the observed changes in the distributions of significant lipids in the 24 h samples, 3 biological replicates of the 24 h time point were imaged simultaneously (Fig. 3). The resulting images show that in each instance across the three sections of the 24 h LSE group, the selectivity of signal for the epidermal edge was a common feature for a broad number of choline rich lipid species. Spots of lipid standards (placed slightly off of the tissue) were included in the imaging experiment and the fact that signals from them were also visible as signals in the tissue added further confirmation to our putative lipid identifications. Species examined in this way were; SM (d18:1/16:0); and PC (16:0/18:1(9Z)).

## Discussion

Generally when employing MSI a number of specifications and tolerances need to be considered. A compromise is usually drawn between peak resolving power with mass accuracy; and the potential for focusing the laser spot diameter with high image resolution, which leads to a loss in sensitivity. Here the use of high mass accuracy and ion mobility separation capabilities enabled separation of isobaric species and precise profiling of the epidermis.

As shown in Additional file 1: Table S1, *m/z* 759.5592 was tentatively identified as an in-source fragment ion of the lipid PC 38:0 and assigned as [M + H – 59 Da]^+^. The ion *m/z* 757.5411 was identified as either PC 38:1 or PE 41:1[M + H – 59 Da]^+^. These ions were indicated as major contributors of variance in the epidermis. It should be noted however that there is still an inherent lack of side-chain specificity when choline-based glycerophospholipids are analyzed in positive ionization mode, as the loss observed is often simply the 184 choline fragment. This means that it is only possible to state that the molecule contains a fatty acid sidechain pool containing a total carbon and unsaturation state *e.g.* 38:0 as stated here. There are a number of possible arrangements for the fatty acids at SN2 *i.e.* 20:0/18:0, 22:0/16:0, 24:0/14:0, etc.

Ceramides in the LSE were visualised mostly as their sodiated adduct [M + Na]^+^. It was possible to aid structural identification of the ceramides in the LSE by incorporating sphingolipid spot standards in the MSI experiment. The tissue ion signals which corresponded with ceramide 6 and glycosylsphingosine spotted standards (as shown in Additional file 1: Figure S2) were important for determining the identifications of sphingoid base ceramides. Such methods for the identification of lipids assisted with spotted standards within the imaging path will be developed further as an alternative to MSMS imaging which only observes products for one ion per image. This could enhance the workflow of untargeted lipidomic investigations where the lipid identification process is often labour intensive; and in some instances biased if the risk of ion carryover between tissue and compound standards is not supervised. Ceramide species vary widely in their carbon chain lengths, extent of unsaturation and in the extent and positioning of hydroxylation in both the fatty acyl and sphingoid moieties [15, 16]. The hope is that, for example, the identification of hydroxyl and non-hydroxylated ceramides can be aided by the identification of characteristic in-source fragment ion patterns using synthetic sphingolipid standards and tissue (characterised by intensity, mass accuracy and drift time) within an imaging experiment. This approach could of course be extended to other lipid classes as well. Spotted standards enabled a combination of mass accuracy and ion mobility drift time values to be matched instantly and independently across the two lipid sources.

Examination of the distribution of the identified phospholipids in the LSE (Figs. 4 and 6), indicates that they are not present in the stratum corneum but at an epidermal junction likely to be the stratum granulosum. Figure 6 shows enlarged MALDI-MS and Haematoxylin and Eosin images of the 24 h sections.

It is reasonable to assume that a human LSE consisting of human keratinocytes, would closely model the lipid activities that likely occur *in-vivo* in native skin. It has been shown previously that a marker of the *stratum corneum,* cholesterol sulphate, could be mapped in skin via negative ion MALDI-MSI [11]. Such lipid features are assumed to be constantly present in the epidermis via highly regulated processes, since it has a role in epidermal desquamation [17, 18]. Here the disposition of TG 54:3 has been shown to be a consistent feature in the stratum corneum in the skin model and this agrees with our own previously published work on the study of *ex-vivo* human tissue by positive ion MALDI-MSI [10]. A central role for TG in epidermal processes may exist. For lipids which are more ubiquitously found across most regions of the tissue, their discrete roles are yet to be determined. Further comparison of Fig. 1 with the previously published data on lipids in the epidermis of *ex-vivo* human skin [10] shows marked consistency in the species observed (but not the signals. In the previous study abundant signals for ions of the form [M + H]^+^ were observed *e.g.* SM 34:1 [M + H]^+^*m/z* 703.6 and PC 34:1 [M + H]^+^*m/z* 760.6, here whilst the same lipid species are observed they are seen as there sodium adducts *i.e.* SM 34:1 as [M + Na]^+^ at *m/z* 725.53 and PC 34:1 as [M + Na]^+^ at *m/z* 782.54. This difference can be accounted for by the extra washing steps to remove salts used in our previous work| [10].

A consistent observation in the MALDI-MS images is that a depletion of phospholipids and glycerosphingolipids occurs across the time-course study. The choline rich lipid features *m/z* 759.5, 757.5, 732.5 and 703.5 are visualised across most of the tissue dermis and epidermis in the early time-points; however at the 24 h time-point, the prominent ion features are localised within the compressed epidermal region, with less signal in the dermis. Phospholipids are constituents of membrane bilayers in cellular organelles of viable cell layers and so the presence of PC species is expected throughout the tissue section. In addition to this, the PC content has been suggested to gradually decrease during keratinocyte differentiation [19]. This is observed as the PC species are not detected in the stratum corneum, although rich in the epidermis. A proportion of the PC content in the epidermis is likely to derive from the subcellular organelles. Phospholipids are also precursor lipids contained within the lamellar bodies, along with glycosphingolipids and free fatty acids. It is known that at the stratum granulosum epidermal interface, the subcellular organelles release their contents into the extracellular spaces [2].

At this 24 h point it could be questioned whether the changes observed in lipid distribution are to do with cell turnover or cell signalling effects; however the histology data shows a significant compression of the epidermis. This is indicative of cell migration towards the outer surface. A reduction of the epidermis surface area could correspond to cell transformation from corneocytes into flattened keratinocyte cells. The MALDI-MS images of the choline head group *m/z* 184 showed the presence of intact tissue across the full thickness of each tissue section, particularly for the 24 h section (data not shown). In all skin models the cells flatten out as the apical surface of the tissue is approached. However, this transition from cubical basal cells to flat granular cells has been shown in the past to occur abruptly in SkinEthic and Episkin cultures [20]; perhaps a factor worth eliminating in the study of cell differentiation in skin equivalent models.

## Conclusions

This work reports identification of lipid changes in LSE which were observed over a time-course experiment by MALDI-MSI. The changes identified in the data are associated with cell differentiation in the dermis and epidermis. In future experiments we will study the effects of topical compounds designed for the treatment of psoriasis and eczema on lipids in LSE. The data reported here will help to differentiate between pharmacodynamic effects arising from the applied compounds and the normal processes of cell differentiation occurring in the LSE in future time course experiments.

## Methods

### Materials

Chemicals used for MALDI matrices and instrument calibration - alpha cyano-4-hydroxycinnamic acid (α-CHCA), aniline, methanol (MeOH), trifluoroacetic acid (TFA), lithium chloride (LiCl) and phosphorus red were purchased from Sigma-Aldrich (Gillingham, UK). Carboxymethylcellulose (CMC) used for embedding samples was purchased from Sigma-Aldrich (Gillingham, UK). For tissue staining protocols haematoxylin, eosin, oil red o, xylene, ethanol (EtOH), triethyl phosphate (TEP) and glycerol gelatine mountant medium were obtained from Sigma-Aldrich (Gillingham, UK). Dulbecco's phosphate-buffered saline and Dulbecco's modification of Eagle's medium, used for tissue washing, were purchased from Invitrogen (Paisley, UK). Lipid standards including sphingomyelin (SM) (d18:1/16:0), glycosphingosine, sphingosylphosphorylcholine (SPC) and ceramide 6 (hexanoyl-D-erythro-D-sphingosine) were purchased from InstruChemie (Delfzyl, Netherlands). C18:1 ceramide (N-oleoyl-D-sphingosine) and PC (16:0/18:1(9Z)) (2-oleoyl-1-palmitoyl-sn-glycero-3-phosphocholine) was purchased from Sigma-Aldrich (Gillingham, UK).

### Ethical Statement

Ethical review, under the Human Tissue Act (HTA) was not required for this work as the types of human tissue that are covered by the HTA are referred to as ‘relevant material’. Relevant material as defined by the HTA includes materials that have come from a human body, whether living or dead, including body parts, organs and human cells. Cell lines, such as those used to construct the living skin equivalents used here are not relevant material (although primary cell cultures are). “Storage of cell lines for research does not require a licence nor does research using cell lines require ethical review”.

### Living skin equivalent samples

Labskin™ living skin equivalent (LSE) samples were provided by Innoven (York UK) after 14 days of development. LSE were delivered as 4.5 cm^2^ ‘surface area inserts’ within transport culture medium. On delivery LSE were partially suspended in LabSkin™ maintenance medium so that the cells were nourished and susequently incubated overnight for 24 h within 5 % CO_2_, 37 °C to normalise their metabolism.

### Skin equivalent time-course experiment protocol

After 24 h incubation the medium was changed for fresh LabSkin™ maintenance medium and LSE samples were incubated for further periods of 4, 6 or 24 h. At the chosen time points samples were removed from the medium washed with Dulbecco’s phosphate buffer solution and left to dry for 5 min at ambient conditions in the holding wells. To quench biological metabolism, the samples were then control frozen to −80 °C using a Grant Asymptote EF600 control freezer and stored at −80 °C until ready for analysis.

### Tissue and lipid standard preparation

For analysis by mass spectrometry imaging and conventional histology 12 μm tissue sections were cut using a cryostat (Leica 2000 UV, Leica Microsystems, Milton Keynes, UK) and were thaw mounted onto conventional glass slides. Sections from each time group were thaw mounted onto the same glass slide, so that they could be directly compared. The mounted sections were carefully washed for 20 s with deionised water to remove any excess salts and other impurities from the skin. Excess water was tapped off and the mounted tissue sections were left to dry briefly in ambient conditions.

Lipid standards each made up at 500 ng/μl (70 % MeOH) including sphingomyelin (d18:1/16:0), ceramide 6 (hexanoyl-D-erythro-D-sphingosine) and PC (16:0/18:1(9Z)) (2-oleoyl-1-palmitoyl-sn-glycero-3-phosphocholine) were spotted (1 μl) at a remote distance to the tissue sections for imaging and profiling correspondence to that which may be present endogenously in the sample. These spots were left to dry in ambient conditions.

### Matrix deposition

For imaging, a matrix solution of 5 mg/ml α-CHCA dissolved in a 70 % MeOH and 0.2 % TFA solution was made up with an equimolar amount of aniline added to the final volume. The matrix was then deposited onto the tissue section surface and over lipid spot standards using a SunCollect™ automated sprayer (KR Analytical, Sandbach, UK).

For MS/MSMS experiments, 100nM of LiCl was dissolved in 70 % MeOH, 0.2 % TFA, with αCHCA being added to the solution to give a concentration of 10 mg/ml. This was then spotted at particular skin regions across the section (1 μl) for profiling; other similar tissue regions were spotted with the non-lithiated matrix for comparison purposes.

### Direct On-tissue and lipid standard MALDI-MS and MS/MS profiling

All analysis were carried out in the positive mode over a mass range of; 50–1000 Da. Ions acquired at 200 laser shots were accumulated at a single position on all MALDI-MS instruments. For MS and MSMS profiling, we adjusted the collision energy (approximately between 2 eV-60 eV) and laser energy (approximately between 185–200 arbitrary units) to produce a decent signal to noise ratio for parent ions and product ions respectively on all MALDI instruments. MS acquisition times varied depending on the focus/purpose of the acquisition. All spectra were post-spectrally calibrated by aligning the measured α-CHCA [M + H] mass with the expected mass (*m/z* 190.050 [M + H]).

MALDI-MS and MSMS spectra were acquired directly from the surface of the *in-situ* skin section samples and lipid spot standards. Analysis was performed using a Waters MALDI HDMS Synapt™ G2 mass spectrometer (Waters Corporation, Manchester, UK) equipped with an Nd: yttrium aluminium garnet (Nd:YAG) laser operated at 1 KHz. The Instrument was calibrated prior to analyses using phosphorus red. The laser spot diameter on the Synapt™ G2 was fixed to 100 μm respectively, sufficient to the width of the epidermis. The peak resolving power was set to Resolution Mode (20,000 FWHM). Using the Synapt™ G2 MS profiles occurred along the epidermis only, or was performed across the full thickness of the tissue. The MS acquisition data was centroided and subjected to post-spectral calibration within the Mass Lynx (Waters Corporation, UK) software tool by performing lock mass of the α-CHCA matrix ion products.

Particularly for the data acquired from the Synapt™ G2, lipids were tentatively identified by accurate mass measurements against values in the Lipidmaps/mMass databases; (mMass lists the masses of possible cationic species for specified lipid compounds). The chemical structure of lipids (as listed on http://www.lipidmaps.org/) that had been assigned with the greatest accuracy (mass tolerance of 5 ppm) were then compared with product ions observed in individual MSMS spectra to confirm identity. MSMS spectra were processed in mMass.

### Multivariate Statistical Analysis and Data processing

Five spectra from the epidermis and/or dermal region respectively for each LSE sample section representing the three time-points (4, 6 or 24 h) were incorporated into a dataset for statistical comparisons (in spectral changes across the time-course per skin layer region). MALDI-MS profiles were attained from the biological specimens at fresh positions. Following post spectral processing (automatic peak detection, post spectral calibration), data lists were exported from Mass Lynx (Waters Corporation, UK) as text files and imported into Marker View software 1.2 (Applied Biosystems/MDS Sciex, Concorde, Ontario, Canada), where it could be formatted into a table. An exclusion list to remove known α-CHCA peaks was applied to the datasets, to remove the influences of the matrix signals when observing relationships of the time-course groups between spectra.

In order to perform principle component analysis (PCA), text files within the table were imported into MatLab® (The Mathsworksinc, Natick, MA, USA) and data was transposed into table format. Once the time-course group classifications had been computationallylabelled, the Eigenvector PLS Toolbox 7.0 was used to perform multivariate statistical analysis on the data in unsupervised modalities. PCA was carried out using data normalisation and mean-centred scaling.

### Direct On-tissue and lipid standard MALDI-MS Imaging

MALDI-IMS-MS images were acquired using a HDMS Synapt™ G2 (Waters Corporation, Manchester, UK) with an Nd:YAG laser, operated at 1 KHz. Prior to the analyses the instrument was calibrated using phosphorus red. Data was acquired at a range of *m/z* 50–1000 in the positive mode. The acquisition of data was performed at a spatial resolution of 50 μm × 50 μm, with the laser energy set to 185 arbitrary units. The data was then processed using the Waters HDI software (Waters Corporation, UK). The average spectra along with the average of ion molecule drift time units were observed simultaneously with spatial abundance of ions within the images. The images were normalised against the total intensity count.

Additional MALDI-MS images were acquired using a modified MALDI-Q-TOF, Q-Star Pulsar-*i* (Applied Biosystems/MDS Sciex, Concord Ontario, Canada) with a NdYVO_4_ laser (Elforlight), operated at 5 kHz. Prior to the analyses the instrument was calibrated using phosphorus red. Data was acquired in positive mode at a range of *m/z* 50*–*1000. The acquisition of data was performed at a spatial resolution of 50 μm × 50 μm using a 40 % laser energy (approximately 2.1 J). The purposes of these images were to serve as technical repeats to the main datasets acquired using the Synapt™ G2 instrument.

### Histology protocols

#### Oil red o staining

12 μm tissue sections were cut using a cryostat (Leica 1850 UV, Leica Microsystems, Milton Keynes, UK) and thaw mounted onto poly-lysine coated glass slides. The mounted slides were firstly immersed into deionised water for 2 min. The tissue slides were then immersed into 100 ml of Oil red O (ORO) working solution (1 g ORO in 60 % triethyl phosphate) for 20 min. After this, the tissue slides were washed in 60 % triethyl phosphate. Slides were mounted using glycerol gelatine mountant medium with a cover slide; and optical images were acquired using an Olympus IX81 microscope.

### Haematoxylin and Eosin (H & E) Staining

Thaw mounted tissue sections were mounted onto poly-lysine coated glass slides as described above. Sections were fixed in 70 % and 90 % EtOH solutions, with each wash lasting for a period of 1 min before being left to air dry. Tissue was then immersed in filtered Harris’s haematoxylin for 30 s and 1 % eosin solution for 30 s. Tissue was rinsed under running tap water after the application of each stain. Dehydration was performed using a series of washes in 50; 70; 80 and 95 % EtOH, each for a period of 2 min. Finally, tissue was immersed in 4 changes of xylene for one minute each. Slides were mounted using DPX mountant and optical images were acquired using an Olympus IX81 microscope.

## Additional file

Additional file 1: Table S1. Table of mass-spectral data produced from profiling the full-thickness of the 24 incubated living skin equivalent tissue; showing tentative lipid identifications (accurate measurements within 5 ppm). Figure S1: a). Drift time mobilogram for lithiated and unlithiated SM (18:1/16:0) showing similar drift-time values for cationic variations of the lipid compound between skin tissue and a spotted lipid standard. b) MALDI mass spectra c). Tandem MS profiling for SM (18:1/16:0) ions in a lipid standard and LSE tissue in the presence of lithium α-CHCA matrix; showing corresponding product ions generated by the collision gas target ion *m/z* 709. Figure S2: MALDI-MS image mapping the ion m/z 725.5 across biological replicates of skin equivalent tissue incubated for 24 h with the time-course experiment. A spot standard of sphingomyelin (d18:1/16:0) has been included to enable putative identification of the ion feature mapped. Images are at a spatial resolution of 50 μm × 50 μm and normalised against the total ion count. (DOCX 2451 kb)

## Abbreviations

MALDI: Matrix assisted laser desorption/ionisation
MS: Mass spectrometry
MSI: Mass spectrometry imaging
IMS: Ion mobility separation
LSE: Living skin equivalent
MSMS: Mass spectrometry mass spectrometry
SM: Sphingomyelin
PC: Phosphatidylcholine
PE: Phosphatidylethanolamine
LPC: Lysophosphatidylcholine
