# Supplementary material for: Lipid changes within the epidermis of living skin equivalents observed across a time-course by MALDI-MS imaging and profiling
Source: Lipids Health Dis. 2015 Aug 5;14:84. doi: 10.1186/s12944-015-0089-z (PMC4525729; doi:10.1186/s12944-015-0089-z)
Supplement: Additional file 1: Table S1. — Table of mass-spectral data produced from profiling the full-thickness of the 24 incubated living skin equivalent tissue; showing tentative lipid identifications (accurate measurements within 5 ppm). Figure S1: a). Drift time mobilogram for lithiated and unlithiated SM (18:1/16:0) showing similar drift-time values for cationic variations of the lipid compound between skin tissue and a spotted lipid standard. b) MALDI mass spectra c). Tandem MS profiling for SM (18:1/16:0) ions in a lipid standard and LSE tissue in the presence of lithium α-CHCA matrix; showing corresponding product ions generated by the collision gas target ion m/z 709. Figure S2: MALDI-MS image mapping the ion m/z 725.5 across biological replicates of skin equivalent tissue incubated for 24 h with the time-course experiment. A spot standard of sphingomyelin (d18:1/16:0) has been included to enable putative identification of the ion feature mapped. Images are at a spatial resolution of 50 μm × 50 μm and normalised against the total ion count. (DOCX 2451 kb) [file 12944_2015_89_MOESM1_ESM.docx]

**Lipid changes within the epidermis of living skin equivalents observed across a time-course by MALDI-MS imaging and profiling.**

Christopher Mitchell^1^, Heather Long^2^, Michael Donaldson^2^, Simona Francese^1^ and Malcolm R Clench^1*^

^1^Biomedical Research Centre, Sheffield Hallam University, Howard Street, Sheffield, United Kingdom, S1 1WB

^2^Stiefel A GSK Company, GlaxoSmithKline, Stockley Park West, Uxbridge, Middlesex,UB1 1BT

^*^Corresponding Author: Professor Malcolm R. Clench, Biomedical Research Centre, Sheffield Hallam University, Howard Street, Sheffield S1 1WB Tel:+44 114 2253054

FAX: + 44 114 225 3066 e-mail: [m.r.clench@shu.ac.uk](mailto:m.r.clench@shu.ac.uk)

S1

Table S1: Table of mass-spectral data produced from profiling the full-thickness of the 24 incubated living skin equivalent tissue; showing tentative lipid identifications (accurate measurements within 5 ppm).

S2


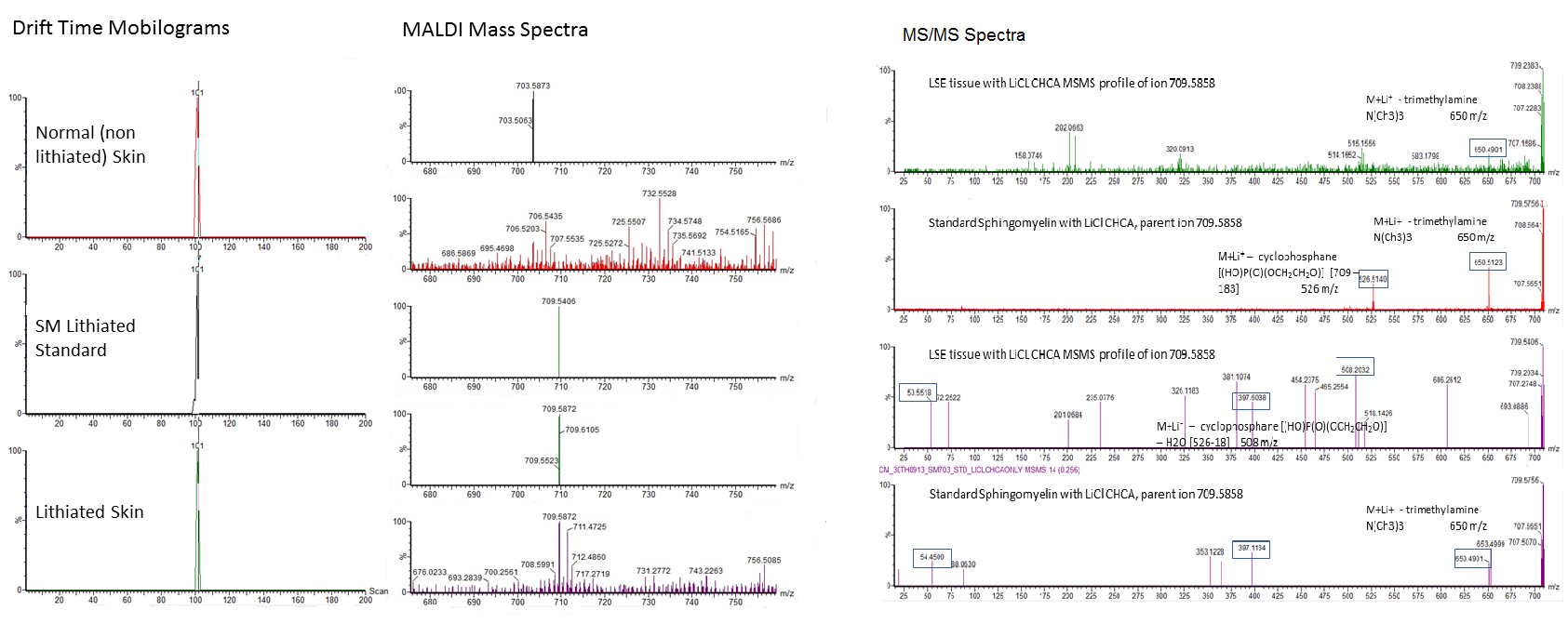


Figure S1: a). Drift time mobilogram for lithiated and unlithiated SM (18:1/16:0) showing similar drift-time values for cationic variations of the lipid compound between skin tissue and a spotted lipid standard. b) MALDI mass spectra c). Tandem MS profiling for SM (18:1/16:0) ions in a lipid standard and LSE tissue in the presence of lithium α-CHCA matrix; showing corresponding product ions generated by the collision gas target ion *m/z* 709.

S3


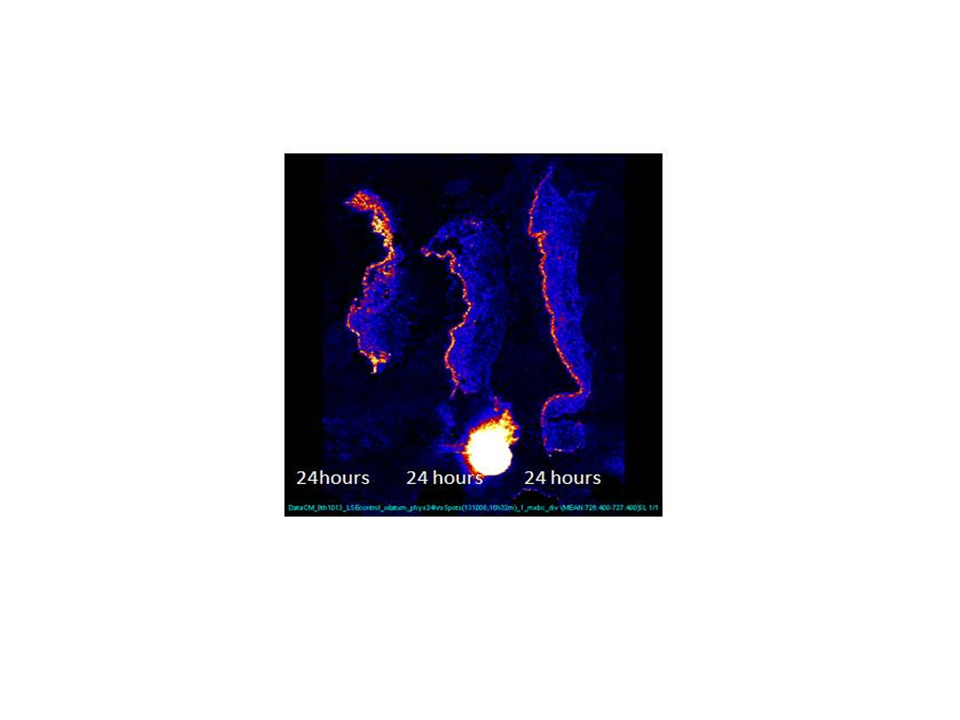


Figure S2: MALDI-MS image mapping the ion m/z 725.5 across biological replicates of skin equivalent tissue incubated for 24hours with the time-course experiment. A spot standard of sphingomyelin (d18:1/16:0) has been included to enable putative identification of the ion feature mapped. Images are at a spatial resolution of 50µm x 50µm and normalised against the total ion count.

S4
